# The Effect of Ursolic Acid on *Leishmania (Leishmania) amazonensis* Is Related to Programed Cell Death and Presents Therapeutic Potential in Experimental Cutaneous Leishmaniasis

**DOI:** 10.1371/journal.pone.0144946

**Published:** 2015-12-16

**Authors:** Eduardo S. Yamamoto, Bruno L. S. Campos, Jéssica A. Jesus, Márcia D. Laurenti, Susan P. Ribeiro, Esper G. Kallás, Mariana Rafael-Fernandes, Gabriela Santos-Gomes, Marcelo S. Silva, Deborah P. Sessa, João H. G. Lago, Débora Levy, Luiz F. D. Passero

**Affiliations:** 1 Laboratory of Pathology of Infectious Diseases (LIM50), Department of Pathology, Medical School of São Paulo University, Av. Dr. Arnaldo, 455. Cerqueira César, São Paulo, 01246-903, SP, Brazil; 2 Laboratory of Clinical Immunology and Allergy (LIM60), University of São Paulo, School of Medicine. Av. Dr. Arnaldo, 455. Cerqueira César, São Paulo, Brazil; 3 Global Health and Tropical Medicine, Instituto de Higiene e Medicina Tropical (IHMT), Universidade Nova de Lisboa, Rua da Junqueira 100, 1349-008 Lisboa, Portugal; 4 Institute of Environmental, Chemical and Pharmaceutical Sciences – Federal University of São Paulo, Rua São Nicolau, 210, 09920-000, Diadema, SP, Brazil; 5 Laboratory of Genetics and Molecular Hematology (LIM31), University of São Paulo, School of Medicine. Av. Dr. Enéas de Carvalho Aguiar, 155. Cerqueira César, São Paulo, Brazil; 6 Department of Pathology, Case Western Reserve University, 2103 Cornell Rd, Cleveland, OH 44106, United States of America; National Center for Cell Science, INDIA

## Abstract

Among neglected tropical diseases, leishmaniasis is one of the most important ones, affecting more than 12 million people worldwide. The available treatments are not well tolerated, and present diverse side effects, justifying the search for new therapeutic compounds. In the present study, the activity of ursolic acid (UA) and oleanolic acid (OA) were assayed in experimental cutaneous leishmaniasis (*in vitro* and *in vivo*). Promastigote forms of *L*. *amazonensis* were incubated with OA and UA for 24h, and effective concentration 50% (EC_50_) was estimated. Ultraestructural alterations in *Leishmania amazonensis* promastigotes after UA treatment were evaluated by transmission electron microscopy, and the possible mode of action was assayed through Annexin V and propidium iodide staining, caspase 3/7 activity, DNA fragmentation and transmembrane mitochondrial potential. The UA potential was evaluated in intracellular amastigotes, and its therapeutic potential was evaluated in *L*. *amazonensis* infected BALB/c mice. UA eliminated *L*. *amazonensis* promastigotes with an EC_50_ of 6.4 μg/mL, comparable with miltefosine, while OA presented only a marginal effect on promastigote forms at 100 μg/mL. The possible mechanism by which promastigotes were eliminated by UA was programmed cell death, independent of caspase 3/7, but it was highly dependent on mitochondria activity. UA was not toxic for peritoneal macrophages from BALB/c mice, and it was able to eliminate intracellular amastigotes, associated with nitric oxide (NO) production. OA did not eliminate amastigotes nor trigger NO. *L*. *amazonensis* infected BALB/c mice submitted to UA treatment presented lesser lesion size and parasitism compared to control. This study showed, for the first time, that UA eliminate promastigote forms through a mechanism associated with programed cell death, and importantly, was effective *in vivo*. Therefore, UA can be considered an interesting candidate for future tests as a prototype drug for the treatment of cutaneous leishmaniasis.

## Introduction

Leishmaniasis is an infectious disease caused by a protozoan belonging to the *Leishmania* genus (Kinetoplastida: Trypanosomatidae) and transmitted to humans through the bite of insect vectors, such as *Lutzomyia* sp. and *Phlebotomus* sp. [1]. In the New World, there are two subgenus and diverse species capable of infecting humans and animals, causing either the visceral or the cutaneous form of leishmaniasis [2]. The cutaneous form of leishmaniasis can be caused by a diverse of *Leishmania* species, therefore a spectrum of clinical signs can be recorded, ranging from a single localized skin lesion, that can heal spontaneously, to multiple ulcerated or non-ulcerated lesions affecting skin and/or mucosa, and these types of lesions frequently require a more complex treatment [3]. However, even considering the broad array of species affecting humans and the clinical types of lesions, the treatment is mainly based on two compounds, antimonial and amphotericin B [4].

Pentavalent antimonials (Sb^V^) are the first choice of treatment for leishmaniasis and were first introduced as trivalent antimonial (Sb^III^) [5]. Although both treatments show toxic effects in patients, which include gastrointestinal intolerance and cardiotoxicity, Sb^V^ shows milder effects [5]. Despite being used as treatment for leishmaniasis for more than a century, the main mechanism of action is poorly understood and in a paradoxical way, the resistance mechanism to Sb^III^ is better known. So far, the most accepted hypothesis is that Sb^V^ acts as a pro-drug and upon administration in humans, a considerable fraction is reduced to its more toxic counterpart Sb^III^, thus causing parasite death. How this reduction occurs is unknown, however, it can be caused through enzymatic reaction or even mediated by plasma or cellular thiols present on the host’s cell phagosomes [6].

Resistance to antimonial drugs was first observed in regions of Bihar—India [7], where reports showed that more than 60% of the patients did not respond to Sb^V^ treatments [8]. The primary resistance mechanism is associated with a decreasing in the concentration of the drug within parasitized cells, that can be mediated by an increase in the efflux of drugs caused by membrane pumps; inhibition of drug activation; or even inactivation of the drug by the parasite or host metabolisms; sequestration and development of bypass mechanisms [9].

Amphotericin B (Amp B) is a polyene antibiotic first isolated in 1955 from *Streptomyces nodosus*. Amp B has replaced antimony treatment in some parts of India due to parasite resistance to antimonial. The treatment with Amp B is effective, since 99.5% of treated patients show no signs of parasites, moreover parasites from relapsing patients did not show resistance to Amp B [10]. In cells, Amp B interacts with the bilipidic layers forming pores, leading to the loss of osmotic regulation capacity of the cells [11]. This interaction leads to the adverse effects associated with Amp B, which include fever, rigors, hypertension/hypotension, hypoxia, renal and gastrointestinal toxicities, limiting its use [12]. As an alternative to decrease the side effects, liposomal and other formulations with Amp B have been made, aiming to reduce serum concentration of the drug, while providing high concentration in the desired area [13]. In the case of liposomal Amp B, a lipid layer is added to the molecule, facilitating its processing in the liver. In spite of that, this type of formulation is an expensive solution if considering that leishmaniasis is more frequent in developing and underdeveloped countries [14]. Based on these findings, the search for less toxic and more effective and less expensive drugs for leishmaniasis treatment is vital.

Plants possess different compounds known as secondary metabolites. These compounds are classified according to their biosynthetic origin, as terpenes, phenolics, and alkaloids, that plants produced in order to facilitate their interaction with the biotic environment [15]. These constituents have a wide array of pharmacologically active compounds and for many poor regions are the unique sources of medicine [16]. In this regard, plants have been used extensively for traditional populations in Latin American to treat leishmaniasis [17, 18], suggesting that compounds present in plants with leishmanicidal effects still are undiscovered by modern medicine. Interesting examples of such compounds are the triterpenoids that comprise more than 20,000 recognized entities, and are biosynthesized in plants through squalene cyclization [19]. The triterpenes can be classified into groups based on their structural skeletons, such as cucurbitanes, cycloartanes, dammaranes, euphanes, friedelanes, holostanes, hopanes, isomalabaricanes, lanostanes, lupanes, oleananes, protostanes, tirucallanes, and ursanes, among others [20]. The diversity of triterpenes is highly associated with their broad range of pharmacological effects, and different studies already showed that these compounds were effective against *Leishmania (Leishmania) major*, *L*. *(L*.*) donovani*, *L*. *(L*.*) infantum*, *L*. *(L*.*) amazonensis*, *L*. *(Viannia) braziliensis*, *L*. *(L*.*) mexicana* and *L*. *(V*.*) panamensis*, suggesting that these classes of compounds present strong leishmanicidal activity [21, 22, 23, 24, 25, 26, 27]. Oleanolic acid (OA) and its isomer, ursolic acid (UA), are triterpenoid compounds that widely occur in nature in the free acid form or as an aglycone precursor for triterpenoid saponins [28]. These triterpenoid acids frequently occur simultaneously because they share similar structural features.

The leishmanicidal effects of OA and UA already were evaluated against promastigote and/or intracellular amastigotes of the following species: *L*. *donovani*, *L*. *major* [29, 22], *L*. *(L*.*) amazonensis* [30], *L*. *(V*.*) braziliensis* [25], and these studies showed that the triterpenes are able to eliminate parasites, suggesting that OA and UA have multispectral action against *Leishmania* sp. In spite of that, there are not works dealing with their possible action mechanisms on *Leishmania* sp., and there is only one study assessing the leishmanicidal effect *in vivo* of a fraction enriched with OA and UA isolated from *Baccharis uncinella* [31]. Considering the multispectral action of triterpenes against *Leishmania* sp. and the absence of studies assessing the therapeutical potential of OA and UA, the present manuscript aimed at analyzing the effect of these triterpenes on *L*. *(L*.*) amazonensis* cultured promastigote and amastigote forms, the mechanism of action of UA on *L*. *(L*.*) amazonensis* as well as the therapeutical effect of UA using the murine model of cutaneous leishmaniasis.

## Methodology

### Ethics statement

All necessary permits were obtained for the described field studies. The State of São Paulo Research Support Foundation (FAPESP), Brazil, and the Brazilian National Research Council (CNPq) provided permits to collect plant material. The site of plant collection is privately owned by São Paulo University, where the authors carried out the plant collection. Procedures involving plant material were in accordance with proper guideline and the field studies did not involve endangered or protected species. *Leishmania (Leishmania) amazonensis* (MHOM/BR/73/M2269) was kindly donated by Prof. Fernando Tobias Silveira, Evandro Chagas Institute, Pará, Brazil. This parasite was maintained as amastigotes in BALB/c mice, acquired from Medical School of São Paulo University, Brazil, that were maintained in appropriated conditions. Animal experiments were carried out using protocols approved by the Ethics Committee for Animal Experimentation of São Paulo University under the number CEP 067/14.

### General experimental procedures

^1^H and ^13^C NMR spectra were recorded, respectively, at 300 and 75 MHz in a Bruker Avance III spectrometer using CDCl_3_ and DMSO-d_6_ (Aldrich) as solvent and internal standard. Silica gel (Merck, 230–400 mesh) and Sephadex LH-20 (Aldrich) were used for column chromatographic separation, while silica gel 60 PF_254_ (Merck) was used for analytical TLC (0.25 mm). Amphotericin B (Cristalia, Brazil) and Glucantime (Sanofi-Aventis, Brazil) were solubilized in sodium chloride 0.9% (w/v).

### Plant material

Leaves of *Petiveria alliaceae* (Phytolaccaceae) were collected in the city of São Paulo, SP, Brazil, in July/2013 and were identified by Dr. Euder G. A. Martins (IB-USP). Voucher specimen has been deposited at Herbarium of Instituto de Botânica-SEMA, São Paulo, SP, Brazil; under reference number PMSP8983.

### Extraction and isolation

Dried and powdered leaves samples of *P*. *alliaceae* (18 g) were exhaustively extracted with hexane (10 X 200 mL) at room temperature. The combined organic fractions afforded, after removal of solvent under reduced pressure, 4.6 g of crude hexane extract. Part of this extract (3.2 g) was subjected to column chromatography (SiO_2_ gel) eluted with a crude solvent gradient of hexane/EtOAc (starting with pure hexane and finishing with pure EtOAc). Using this approach, were obtained 14 fractions (A–N). As fraction D (412 mg) displayed *in vitro* antileishmanial activity, this material was purified by column chromatography over Sephadex LH-20 with MeOH as the eluent. This second purification step afforded 111 mg of a mixture of triterpene acids. Purification by semi-preparative RP-18 HPLC, eluted with MeOH:H_2_O 95:5 (flow rate at 5 mL/min and detector at λ = 218 nm) yielded ursolic (31 mg) and oleanolic (45 mg) acids.

Ursolic acid (99.4% of purity determined by HPLC). ^1^H NMR (300 MHz, CDCl_3_ + DMSO-d_6_) δ_H_: 4.89 (br s, H-12), 4.06 (br s, H-3), 1.87 (d, *J* = 3.4 Hz, H-18), 1.68 (s, H-23), 1.61 (s, H-27), 1.52 (s, H-25), 1.31 (s, H-26), 0.80 (br s, H-30), 0.63 (br s, H-29), 0.58 (s, H-24). ^13^C NMR (75 MHz, CDCl_3_ + DMSO-d_6_) δ_C_: 178.7 (C-28), 138.6 (C-13), 125.0 (C-12), 77.3 (C-3), 55.4 (C-5), 53.0 (C-18), 47.3 (C-9), 47.5 (C-17), 42.1 (C-14), 40.5 (C-8), 40.2 (C-19), 40.0 (C-10), 39.7 (C-22), 39.6 (C-7), 39.5 (C-21), 39.4 (C-23), 38.8 (C-15), 36.8 (C-1), 30.7 (C-4), 33.2 (C-20), 28.7 (C-2), 28.0 (C-29), 27.4 (C-16), 24.3 (C-27), 23.7 (C-11), 21.6 (C-30), 17.5 (C-6), 17.3 (C-26), 16.5 (C-25), 15.7 (C-24).

Oleanolic acid (99.9% of purity determined by HPLC). ^1^H NMR (300 MHz, CDCl_3_) δ_H_: 5.16 (br s, H-12), 3.07 (m, H-3), 2.73 (d, *J* = 4.0 Hz, H-18), 1.47 (s, H-23), 1.22 (s, H-27), 0.88 (s, H-25), 0.69 (s, H-26), 1.03 (s, H-30), 0.82 (s, H-29), 0.78 (s, H-24). ^13^C NMR (75 MHz, CDCl_3_) δ_C_: 180.0 (C-28), 143.8 (C-13), 121.9 (C-12), 78.0 (C-3), 55.1 (C-5), 47.4 (C-9), 46.0 (C-19 and C-17), 41.6 (C-14), 41.0 (C-18), 39.2 (C-8), 38.6 (C-4), 38.4 (C-1), 36.8 (C-10), 33.8 (C-21), 33.0 (C-29), 32.6 (C-7), 32.3 (C-22), 30.6 (C-20), 28.1 (C-23), 27.5 (C-15), 27.0 (C-2), 25.8 (C-27), 23.5 (C-30), 23.2 (C-16), 22.9 (C-11), 18.2 (C-6), 16.9 (C-26), 15.7 (C-24), 15.2 (C-25).

### Parasites

*L*. *(L*.*) amazonensis* parasite (MHOM/BR/73/M2269) was kindly provided by Prof. Dr. Fernando T. Silveira from the criobank of “Leishmaniasis Laboratory Prof. Dr. Ralph Laison”, Department of Parasitology, Evandro Chagas Institute, Ministry of Health, Belém, Pará, Brazil. The parasite was identified using monoclonal antibodies and isoenzyme electrophoretic profiles at the Leishmaniasis Laboratory of the Evandro Chagas Institute (Belém, Pará state, Brazil). This parasite was grown in Roswell Park Memorial Institute-1640 medium—RPMI 1640 (Gibco^®^, Life Technologies, Carlsbad, CA, USA), supplemented with 10% heat-inactivated fetal bovine serum, 10 μg/mL of gentamicin, and 1,000 U/mL of penicillin (R10) at 25°C. Promastigote forms in the stationary phase were used.

### Animals

Six to eight weeks old female BALB/c mice were obtained from Medical School of São Paulo University. This study was carried out in strict accordance with the recommendations in the Guide for the Care and Use of Laboratory Animals of the Brazilian National Council of Animal Experimentation (http://www.cobea.org.br). The protocol was approved by the Committee on the Ethics of Animal Experiments of the Institutional Animal Care and Use Committee at the Medical School of São Paulo University (CEP 322/12). For all experimental procedure mice were anaesthetized with tiopental (1mg/200 μL).

### Evaluation of anti-promastigote effect of OA and UA

Promastigote forms of *L*. *(L*.*) amazonensis* (2x10^7^ promastigotes/mL) were incubated in 96-well culture plate in R10 medium with OA or UA in a range of 100 to 4x10^-1^ μg/mL. The standard drug Amp B was added in culture in the range of 1.0 to 4x10^-3^ μg/mL and Miltefosine in the range of 100 to 4x10^-1^ μg/mL. Negative control group was cultivated in medium and DMSO as vehicle solution (never exceeding 1% v/v). The parasites were incubated for 24h at 25°C. Then, the plate was washed with 200 μL of sodium chloride 0.9% (w/v) three times with centrifugation at 3000 rpm, 10 min at 4°C, followed by addition of MTT (3-(4,5-dimethylthiazol-2-yl)-2,5-diphenyltetrazolium bromide) (4 mg/mL). Four hours later, 50 μL of 10% sodium dodecyl sulfate (SDS) was added to each well. The plates were further incubated for 18h and read in ELISA reader at 595 nm. Effective concentration 50% (EC_50_) was estimated using Graph Pad Prism 5.0 software.

### Peritoneal macrophages culture and cytotoxicity assay

Approximately 2x10^5^ peritoneal macrophages from BALB/c mice were cultured in R10 medium with UA or amphotericin B (100.00 to 0.01 μg/mL). As negative control, macrophages were cultivated in medium and DMSO as vehicle solution (never exceeding 1% v/v). After 24h, cell viability was analyzed by MTT method. Cytotoxic concentration 50% (CC_50_) was estimated with Graph Pad Prism 5.0 software was used for plotting and statistic analysis. The index of selectivity was obtained through the expression:

$$SI=\;\frac{\;CC_{50}}{EC_{50}}$$

### Ultra-structural alterations induced by UA in *L*. *(L*.*) amazonensis* promastigotes

Promastigote forms of *L*. *(L*.*) amazonensis* (2x10^7^ promastigotes/mL) were incubated in 96-well culture plate in R10 medium with the EC_50_ of UA for 24h, at 25°C. Control group was cultivated with medium and vehicle solution DMSO (never exceeding 1% v/v). The plate was centrifuged at 3000 rpm, 4°C, 10 min and washed three times with 200 μL of sodium chloride 0.9%. Then the pellets were ressuspended in glutaraldehyde 2% and incubated at 4°C, during 60 min. Parasites were post-fixed in 1% osmium tetroxide, and these materials were stained and block staining in 1% aqueous uranyl acetate overnight, dehydrated using alcohol. Then, samples were embedded in a polyester resin, thin sectioned with a LKB ultratome, double-stained by uranyl acetate and lead citrate (Ladd Research Industries), and examined with a Jeol 1010 (Tokyo, Japan) transmission electron microscope (TEM).

### Studies of cell death mechanism

In order to evaluate the pathway of programed cell death triggered by UA, modification in the pattern of phosphatidylserine, caspase 3/7 protease activity, genomic DNA fragmentation, and mitochondrial membrane potential were evaluated. To perform all these sets of experiments, promastigote forms of *L*. *(L*.*) amazonensis* (2x10^7^ promastigotes/mL) were incubated in 96-well culture plate in R10 medium with the EC_50_ of UA for 24h, at 25°C. In addition, promastigote forms of *L*. *(L*.*) amazonensis* were also incubated with EC_50_ of hydrogen peroxide (5.8 μM) as an apoptosis inducer [32]. Control group was cultivated with medium and vehicle solution DMSO. After incubation, plates were centrifuged at 3000 rpm, 4°C, 10 min and washed three times with PBS.

To analyze exposure of phosphatidylserine on membrane, parasites were stained with Annexin V (FITC) and PI according to manufacturer’s instructions (LifeTech). *L*. *(L*.*) amazonensis* promastigotes treated with EC_50_ of H_2_O_2_ were single stained with Anexin V (FITC) or PI and were used for cytometer adjusts. The samples were acquired in Fortessa II cytometer, and around 10^6^ events were recorded. The data were analyzed in FlowJo 10, and parasites were characterized: 1) viable: Annexin V^-^/Pi^-^; 2) early stages of apoptosis: Annexin V^+^/Pi^-^; 3) late stages of apoptosis: Annexin V^+^/Pi^+^ and 4) other type of cell death: Annexin V^-^/Pi^+^.

To analyze the involvement of caspase in cell death, caspase-3/7 protease activity was measured using the Apo-1 homogenous caspase-3/7 activity assay kit (Promega, USA). The assay was done according to the manufacturer’s instructions. In this set of experiment, parasites were incubated with caspase substrate for 120 min and increase in the fluorescence was an indicative of substrate cleavage. Plates were fluorometrically read at excitation and emission wavelengths of 485 and 530 nm, respectively.

To analyze the pattern of DNA fragmentation genomic material from pellets of treated and control parasites was purified using commercial kits (LifeTechnologies). The amount of DNA was quantified, adjusted to 50ng and applied into 1.5% agarose gel plus 0.001% of gel red (Uniscience, Brazil). DNAs were subjected to electrophoresis in Tris-Borate-EDTA buffer for 2 h at 100V. DNA was then visualized under UV light.

The mitochondrial membrane potential of treated and non-treated promastigotes was investigated using the JC-1 (5,5’,6,6’-tetrachloro-1,1’,3,3’-tetraethylbenzimidazolcarbocyanine iodide) marker, according to the manufacturer´s instruction (B&D, USA). Treated and control parasites were incubated with JC-1 and Hoeschst solutions for 15min, 25°C. Parasites were analyzed by High Content Analysis ImageXpress Micro high content screening system (Molecular Devices, Sunnyvale, CA), and at least nine sites per well and three wells per treatment were acquired. The presence of JC-1 aggregates was determined using the transfluor MetaXpress software.

### Macrophage infection and treatments, nitric oxide and cytokine determination

Peritoneal macrophages from BALB/c mice (2x10^5^ macrophage) were cultivated in round cover slips in 24-well plate, followed by infection with *L*. *(L*.*) amazonensis* promastigotes at a ratio of 10 parasites per 1 peritoneal macrophage. Plates were incubated at 5% CO_2_ at 35°C. After 24h of culture, UA (0.1, 1.0 and to 10.0 μg/mL) and amphotericin B (0.1 μg/mL) were added. After 24 and 72h supernatants were collected and stored at −80°C for nitric oxide (Life Technologies, USA) and IL-10, IL-12 and TNF-α (B&D Bioscience, USA) quantifications, according to manufacturer’s instructions. Round cover slips from each experimental time-point were dried at room temperature, fixed in methanol, and stained by Giemsa. The Infection Index (II) was then estimated according to Passero et al 2015 [33], following the expression:

$$II=\;\%\;Infected\;macrophages\;\times \;\frac{Internalized\;amastigote\;forms}{Macrophage}$$

### Infection and experimental treatment

Twenty male BALB/c mice were subcutaneously infected into the right hind footpad with 10^6^ promastigote forms of *L*. *(L*.*) amazonensis* and five BALB/c mice received only sodium chloride 0.9% (w/v) under the same route (healthy group). Twenty days after infection, *L*. *(L*.*) amazonensis*-infected BALB/c mice were divided into four groups: group # 1 was injected with the dose of 1.0 mg of UA by kg of body weight (mg/kg); group # 2 was injected with 2.0 mg/kg of UA, group # 3 was injected with 100.0 mg/kg of Glucantime [34], groups # 4 (only infected—control group) was injected with PBS solution. Group # 5 was constituted by animals that received only vehicle solution (PBS control). Glucantime, UA and vehicle solution were injected intralesionally four times, once a day, at intervals of 2 days each. The physical conditions of the animals were monitored once a week. Two weeks after the last injection, animals were anaesthetized with thiopental and sacrificed by cardiac punction. There was no dead prior to the endpoint and all animals were euthanized to analyze skin parasitism.

### Clinical course of lesion development and determination of parasite burden in skin

The development of lesions in infected and treated groups was measured weekly after infection. The size of lesion was obtained from the difference between infected and uninfected footpads.

The parasite load in the skin was determined using the quantitative limiting-dilution assay, as previously described [35]. Briefly, fragments from infected footpad of different groups were aseptically excised and homogenized in Schneider’s medium. The skin suspensions were subjected to 12 serial dilutions with four replicate wells. The number of viable parasites was determined based on the highest dilution that promastigotes could be grown after 10 days of incubation at 25°C. Biopsies of the skin inoculation site from treated and non-treated groups were collected and fixed in buffered 5% formalin for histopathological studies.

### Statistical analysis

The results were expressed as the mean ± standard deviation of three independent experiments and the nonparametric Mann-Whitney U test was used to compare results among groups. Differences were considered statistically significant at 5% significance level (p<0.05). GraphPad Prism 5 (GraphPad Software, Inc., La Jolla, CA, USA) was used to analyze the results.

## Results

### Identification of oleanolic and ursolic acids

**Fig 1:**
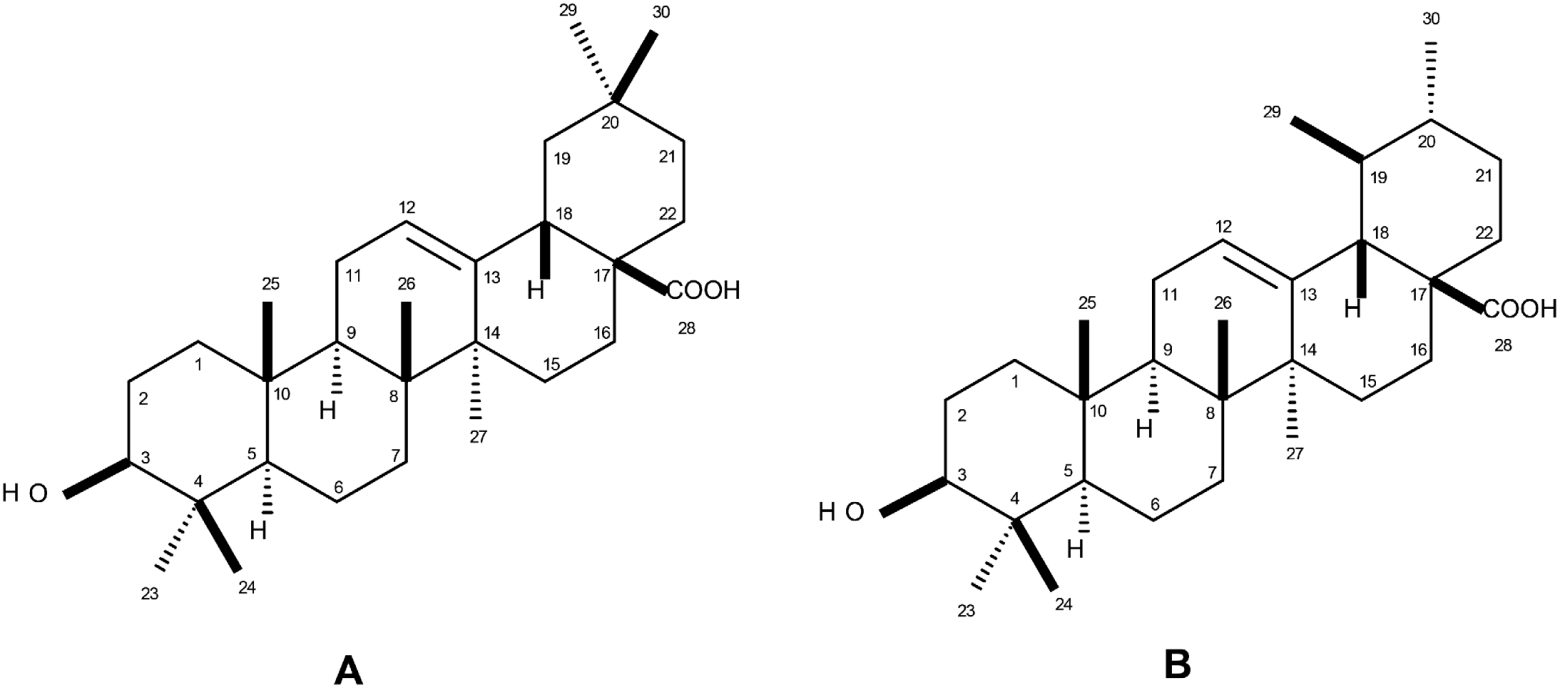
Structures of oleanolic (A) and ursolic acids (B).

Analysis of ^1^H NMR spectra of purified compound displayed signals of olefin and carbinol hydrogens, respectively, at δ 5.16/4.89 (H-12) and 3.07/4.06 (H-3). These signals, associated with the presence of doublets at δ 2.73 (*J* = 4.0 Hz) and 1.87 (*J* = 3.4 Hz) are characteristic of H-18 of oleanane and ursane triterpenoid, respectively [36]. ^13^C NMR spectra showed signals attributed to olefin carbons at δ 121.9/125.0 (C-12), 143.8/138.6 (C-13), 78.0/77.3 (C-3) and 180.0/178.7 (C-28). The presence of these signals associated to those attributed to the methyl groups C-23 (δ 28.1/39.4), C-24 (both at δ 15.7), C-25 (δ 15.2/16.5), C-27 (δ 24.3), C-29 (δ 33.0/28.0), and C-30 (δ 23.5/21.6), allowed the identification of oleanolic and ursolic acids [37], as indicated in Fig 1.

### UA eliminates promastigote and amastigote forms of *L*. *(L*.*) amazonensis* and did not present toxicity for BALB/c peritoneal macrophages

Promastigotes forms of *L*. *(L*.*) amazonensis* were treated with UA and OA as well as two standard drugs, amphotericin B and Miltefosine (Table 1). Results showed that UA eliminated *L*. *(L*.*) amazonensis* promastigote in a dose-dependent manner, showing an EC_50_ of 6.2 ± 1.8 μg/mL; on the other hand, OA presented only a marginal effect on promastigote forms, eliminating around 14% of parasites with 100 μg/mL. Both Amp B and Miltefosine eliminated promastigote forms in a dose-dependent manner, and the EC_50_ were 1.5 ± 0.5 μg/mL and 5.9 ± 1.8 μg/mL, respectively (Table 1). Regarding macrophage toxicity, it was observed that UA and amphotericin B presented CC_50_ of 56.1 and 57.3 μg/mL, while miltefosine was 34.4 μg/mL. These data suggest that UA and standard drugs were not toxic for macrophages, being the selectivity index of UA intermediated among miltefosine and amphotericin B (Table 1).

**Table 1:** EC_50_, CC_50_ and SI induced by OA, UA, amphotericin B and Miltefosine against *L*. *(L*.*) amazonensis* promastigotes and macrophages.

	EC_50_ (μg/mL)	CC_50_ (μg/mL)	SI
Oleanolic acid	> 100	> 100	ND
Ursolic acid	6.2 ± 1.8	56.1 ± 6.1	9.1
Amphotericin B	1.5 ± 0.5	57.3 ± 9.3	38.2
Miltefosine	5.9 ± 1.8	34.4 ± 4.6	5.8

### Ultra-structural alterations induced by UA in *L*. *(L*.*) amazonensis* promastigotes

**Fig 2:**
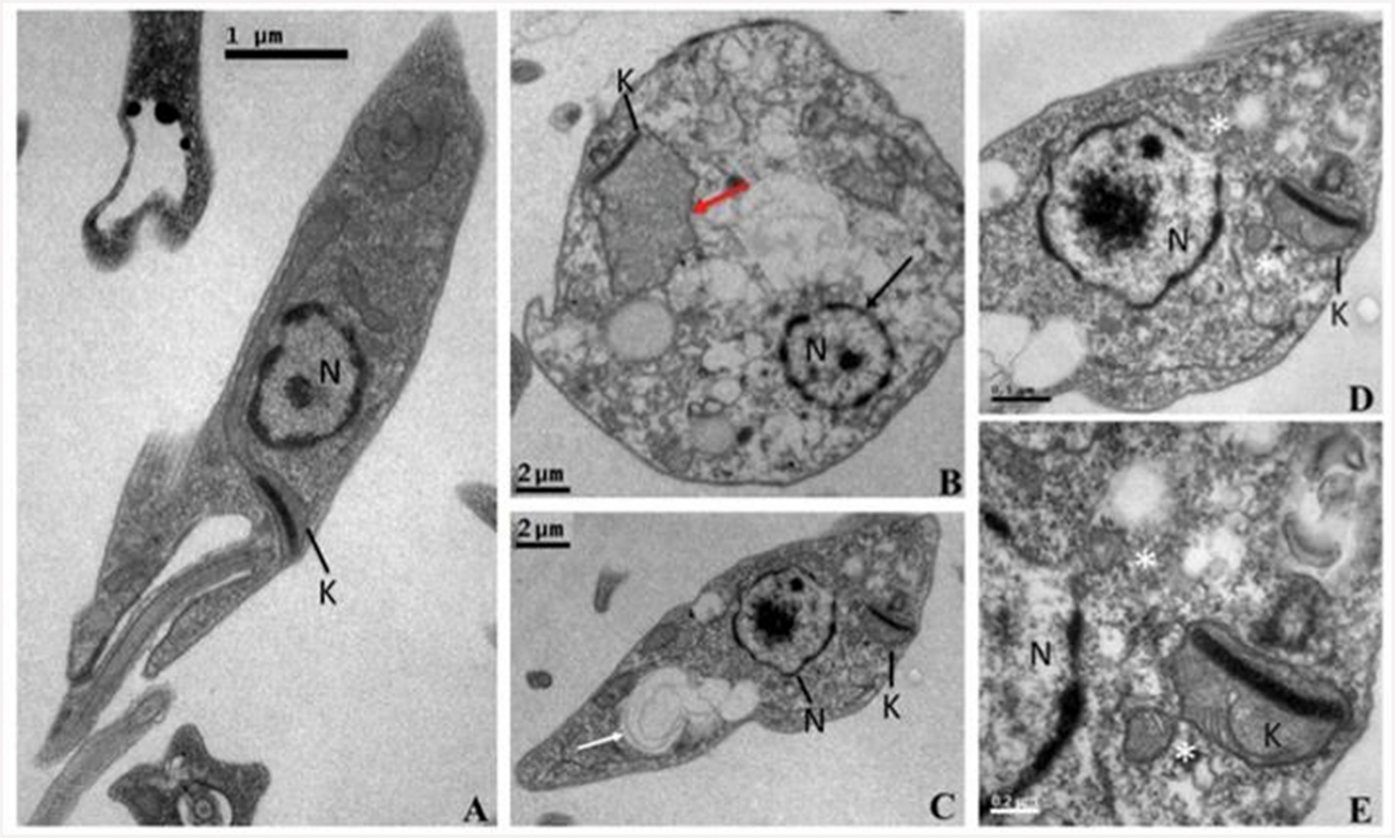
Ultrastructural changes of *L*. *(L*.*) amazonensis* promastigote induced by *in vitro* treatment with UA. Images of cultured promastigote forms before (**A**) and after treatment (B, C, D and E) with UA EC_50_ were captured by transmission electron microscopy. (N)–Nucleus, (K)–Complex kinetoplast-Mitochondria; red arrow: mitochondrion swelling; white arrow: membrane-containing vacuoles; white asterisk: blebs on nucleus and mitochondria.

Concerning parasite ultrastructure, control promastigotes (Fig 2A) presented typical ultrastructural characteristics: a fusiform shape with intact cell membrane, an apical extracellular flagellum, cytoplasm presenting normal morphology and well-preserved intracellular structures. The nucleus showed nucleolus at central position and peripheral chromatin, the mitochondria extends posteriorly containing the kinetoplast apically localized. On the other hand, parasites treated with UA EC_50_ presented major ultrastructural changes. Treated promastigotes showed round-shape morphology, cytoplasm vacuolization and disorganization (Fig 2B) and autophagosome-like structure containing internal membranes (Fig 2C, white arrow). The complex mitochondria-kinetoplast acquired a swelling-shape (Fig 2B, red arrow) and exhibited blebs (Fig 2D and 2E, white asterisks). Nuclear alteration suggests loss of compact shape (Fig 2B, black arrow), and chromatin was condensed (Fig 2C and 2D); blebs at nuclear envelope were detected (Fig 2D and 2E, white asterisks).

### Studies of cell death mechanism

**Fig 3:**
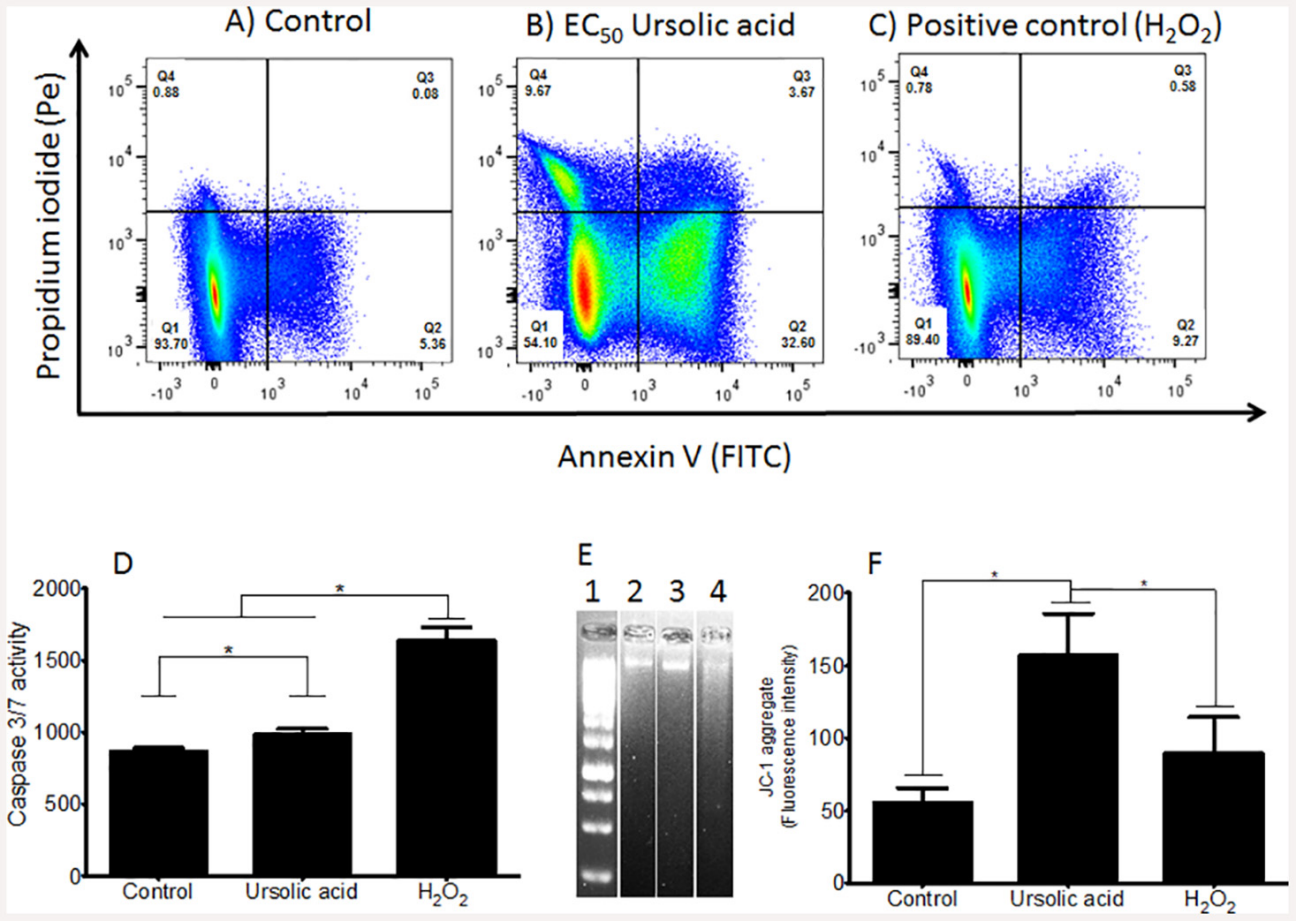
The mechanism of parasite death was investigated after *in vitro* treatment with UA. By flow cytometry the pattern of Annexin V and PI staining was analyzed in non-treated promastigotes (A), UA-treated promastigotes (B) and H_2_O_2_-treated promastigotes (C); and it was characterized as “viable cells” in Q1 (Annexin V^-^/PI^-^), “early apoptosis stage” in Q2 (Annexin V^+^/PI^-^), “late apoptosis stage” in Q3 (Annexin V^+^/PI^+^) and “cellular death/necrosis” in Q4 (Annexin V^-^/PI^+^). D—Activity of caspase 3/7 was investigated in UA-treated parasites. E—Aspect of nuclear DNA from control parasites (lane 2), UA-treated parasites (lane 3) and H_2_O_2_-treated parasites, molecular marker is represented in lane 1; F—JC1 aggregates were analyzed in control, UA—and H_2_O_2_—treated parasites.

Promastigote forms of *L*. *(L*.*) amazonensis* were treated with UA EC_50_ and H_2_O_2_ (positive control of apoptosis) and 24h later the mechanisms of cell death were evaluated. Control promastigotes (Fig 3A) presented 93.7% viability (Anexin V^-^/PI^-^, quadrant 1 –Q1), a small frequency of parasites was dead, being 5.4% of the parasites in early apoptosis (Anexin V^+^/PI^-^, Q2), 0.087% in late apoptosis (Anexin V^+^/PI^+^, Q3) and 0.9% were necrotic (Anexin V^-^/PI^+^, Q4). In contrast, *L*. *(L*.*) amazonensis* treated with UA EC_50_ (Fig 3B) showed an important decrease of viable parasites (54.1% Annexin V^-^/PI^-^, Q1). A significant population of UA—treated promastigotes was in early apoptosis (33% Annexin V^+^/PI^-^, Q2), 3.7% of parasite population was in late apoptosis (Anexin V^+^/PI^+^, Q3) and 9.7% of the parasite was necrotic (Annexin V^-^/PI^+^, Q4) (p < 0.05). After treatment with H_2_O_2_, the majority of the parasites were viable (89.40% Annexin V^-^/PI^-^, Q1), however the frequency of Annexin V^+^/PI^+^ population (9.30% in Q2) was two times higher than control parasites (p < 0.05). In addition, 0.58% of parasites were in late apoptosis (Annexin V^+^/PI^+^, Q3) and 0.80% was necrotic (Annexin V^-^/PI^+^, Q4).

Fig 3D shows that UA-treated parasites presented a slight significant increasing in the activity of caspase 3/7 in comparison with control (p < 0.05), and genomic DNA did not show aspect of fragmentation (Fig 3E, lane 2). On the other hand, H_2_O_2_ –treated parasites presented high activity for caspase 3/7 compared to control and UA—treated parasites (Fig 3D), and DNA showed fragmented (Fig 3E, lane 4). As indicated in Fig 3F, mitochondria from parasites treated with UA presented significantly more JC-1 aggregate in comparison with control parasites and it was higher compared to H_2_O_2_ –treated parasites (p < 0.05).

### UA eliminates intracellular amastigotes and triggers NO production by treated cells

**Fig 4:**
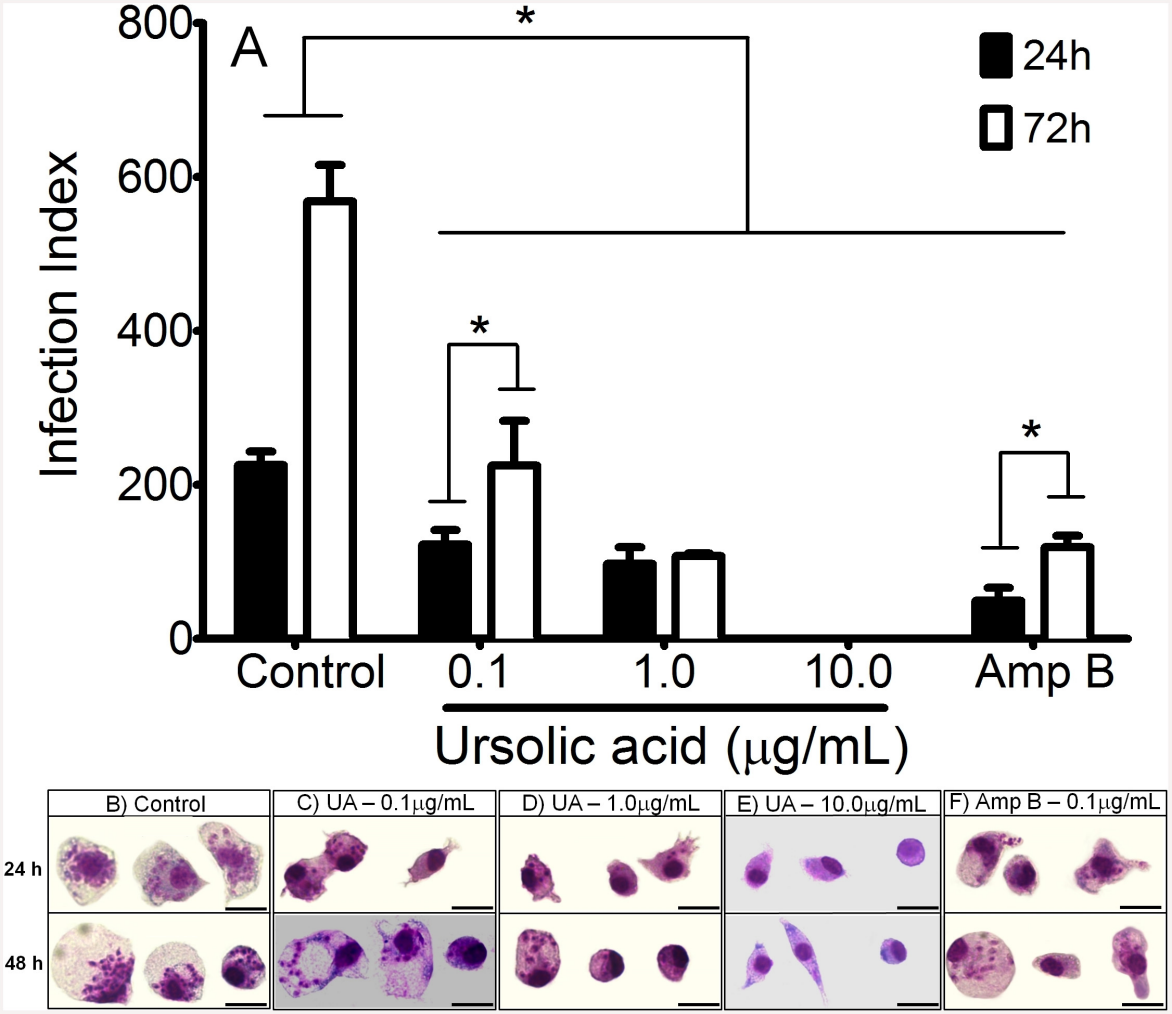
Infection Index (II) of *L*. *(L*.*) amazonensis*-infected peritoneal macrophages submitted to different treatments (A). Peritoneal macrophages were infected with promastigote forms of *L*. *(L*.*) amazonensis* and treated for 24 and 72h with UA or amphotericin B (Amp B– 0.1 μg/mL). Illustration of control infected macrophages (B), infected cells treated with 0.1 μg/mL (C), 1.0 μg/mL (D) and 10.0 μg/mL (E) of UA as well as 0.1 μg/mL of amphotericin B (F) at 24 and 72h. Results are represented by mean and standard deviation of three independent experiments and three replicates per sample. * (p<0.05) indicate significant differences.

After 24h and 72h of UA treatment, alterations in the Infection Index (II) were observed. Infected peritoneal macrophage treated with 0.1 and 1.0 μg/mL of UA decreased significantly (p<0.05) the II in comparison with their respective control (Fig 4A, 4B, 4C and 4D), in addition infected peritoneal macrophages treated with 10.0 μg/mL of UA did not present evidence of amastigote forms in cultures of 24 and 72h of treatment, as shown in Fig 4A and 4E. Macrophages treated with 0.1 μg/mL amphotericin B decreased significantly the II compared to control (Fig 4A and 4F) at 24 and 72h (p<0.05). At 72h, infected peritoneal macrophages treated with 0.1 μg/mL of UA and amphotericin B presented higher II in comparison with 24h of treatment.

**Fig 5:**
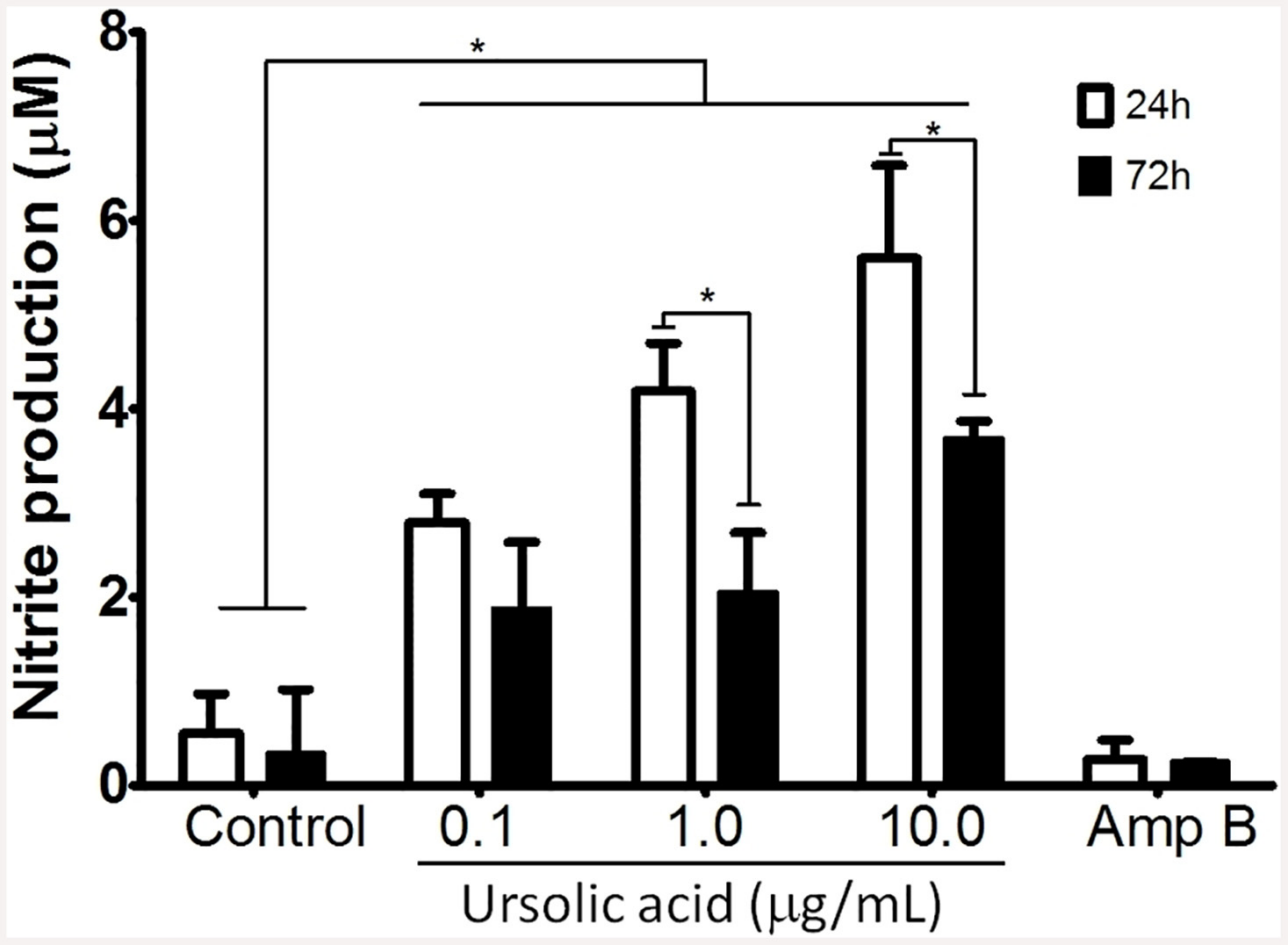
Nitrite levels were evaluated in supernatant of *L*. *(L*.*) amazonensis*-infected peritoneal macrophages submitted to different treatments. Peritoneal macrophages were infected with promastigote forms of *L*. *(L*.*) amazonensis* and treated for 24 or 72h with UA or amphotericin B (Amp B– 0.1 μg/mL). Results are represented by mean and standard error of three independent experiments and three replicates per sample. * (p<0.05) indicates significant differences.

At 24 and 72h of treatment (Fig 5), levels of NO in the supernatant of UA-treated macrophages were significantly higher in comparison to its respective control group (p<0.05). However, in comparison with 24h of treatment, macrophages treated with 1.0 and 10.0 μg/mL of UA for 72h presented a significant reduction of NO levels (p<0.05). Amphotericin B—treated macrophages produced similar amount of NO to control group at 24 and 72h. IL-12, IL-10 and TNF-α cytokines were not detected.

### UA presents therapeutic action in experimental cutaneous leishmaniasis

**Fig 6:**
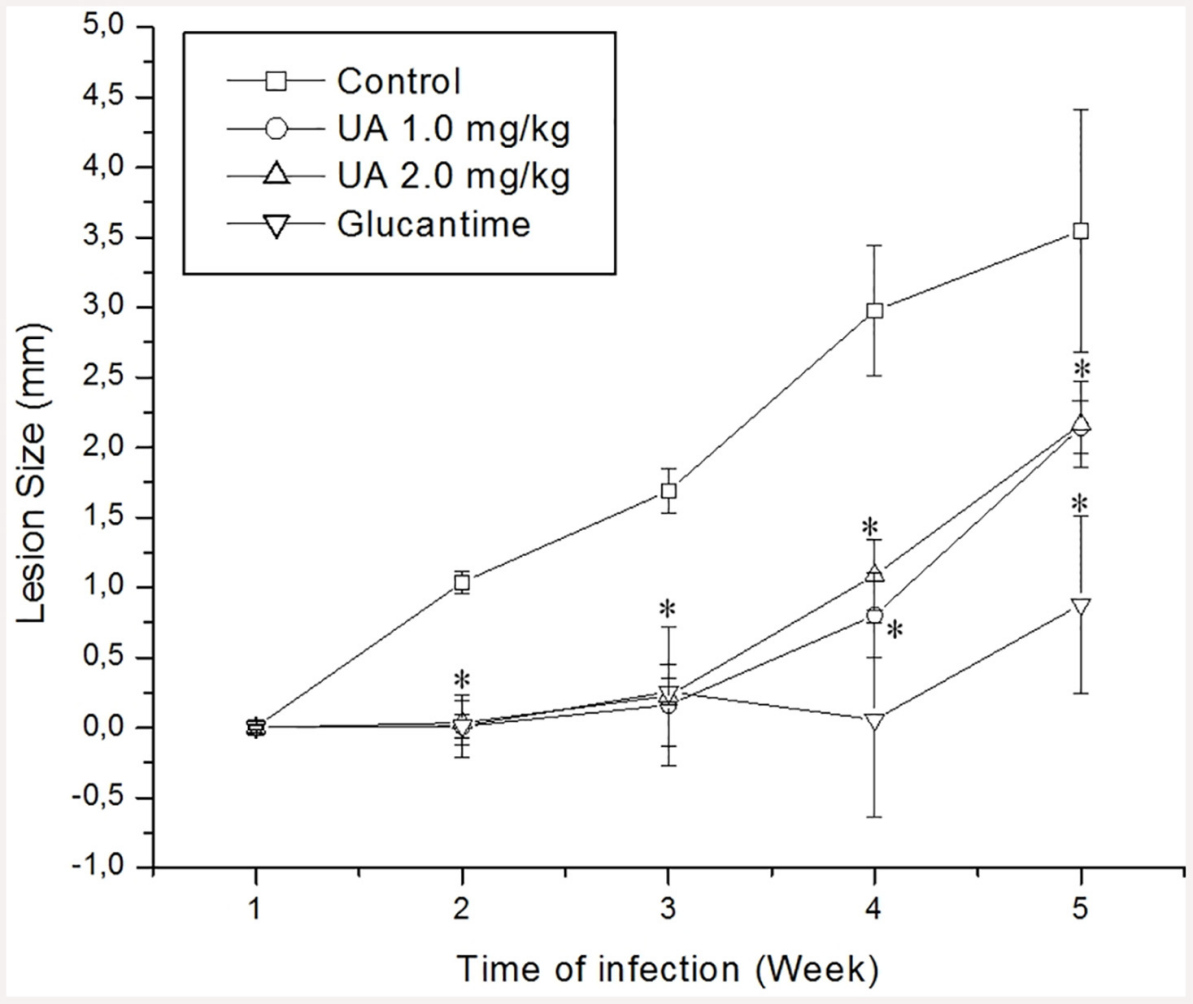
BALB/c mice were infected with promastigote forms of *L*. *(L*.*) amazonensis* and treated with 1.0 and 2.0 mg/kg of UA or 100.0 mg/kg of Glucantime (Glu). The development of the disease was monitored for 5 weeks through weekly measurement of lesions. Results are represented by mean and standard error of three independent experiments, containing 5 animals per group. * (p<0.05) indicates significant differences when compared treated groups vs infected, non-treated group.

Animals treated with UA (1.0 and 2.0 mg/kg) or Glucantime (100.0 mg/kg) presented decreased lesion size in comparison to infected control (p<0.05) from 2 to 5 weeks post infection, as indicated in Fig 6. At 5 weeks post-infection animals treated with 100.0 mg/kg of Glucantime presented lower lesion size in comparison with UA-treated groups (p<0.05). No important differences were observed in lesion size between mice treated with different UA doses (Fig 6).

**Fig 7:**
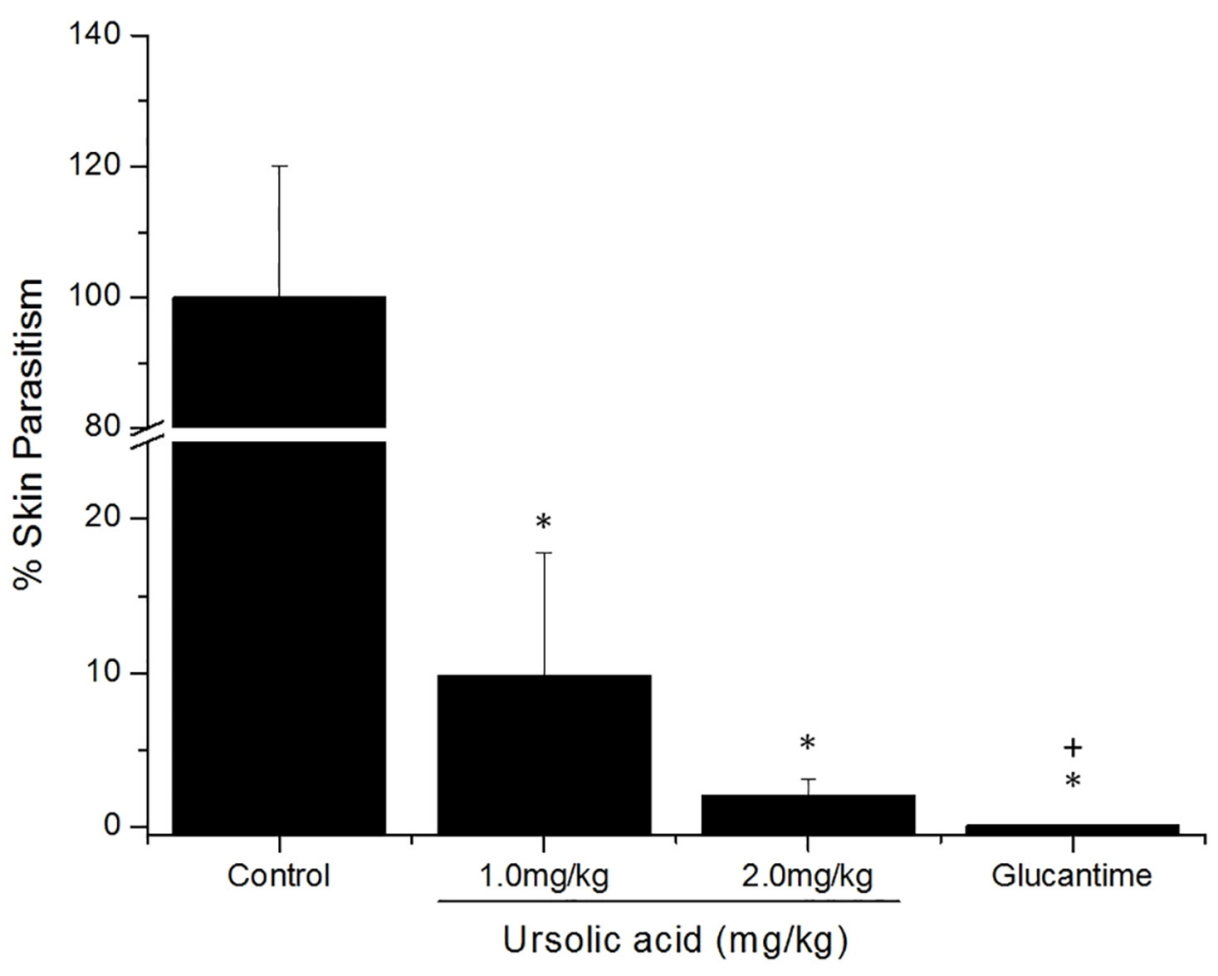
Parasite inhibition after treatment. Footpad lesions of control mice and of mice treated with of UA (1.0 mg/kg or 2.0 mg/kg) or Glucantime (100.0 mg/kg). Results are represented by mean and standard error of three independent experiments containing five animals per group. * and + (p<0.05) indicate significant differences when compared non-treated vs treated mice and, Glucantime vs UA treated mice, respectively.

Regarding skin parasitism, BALB/c mice treated with 1.0 and 2.0 mg/kg of UA showed lesser skin parasitism in comparison with infected control (p<0.05), representing a decreasing of parasitism of 91 and 98%, respectively. Glucantime eliminated 99.9% parasites (Fig 7). The efficacy of 2.0 mg/kg UA in eliminating parasites during mice treatment was similar to parasite reduction achieved with Glucantime.

**Fig 8:**
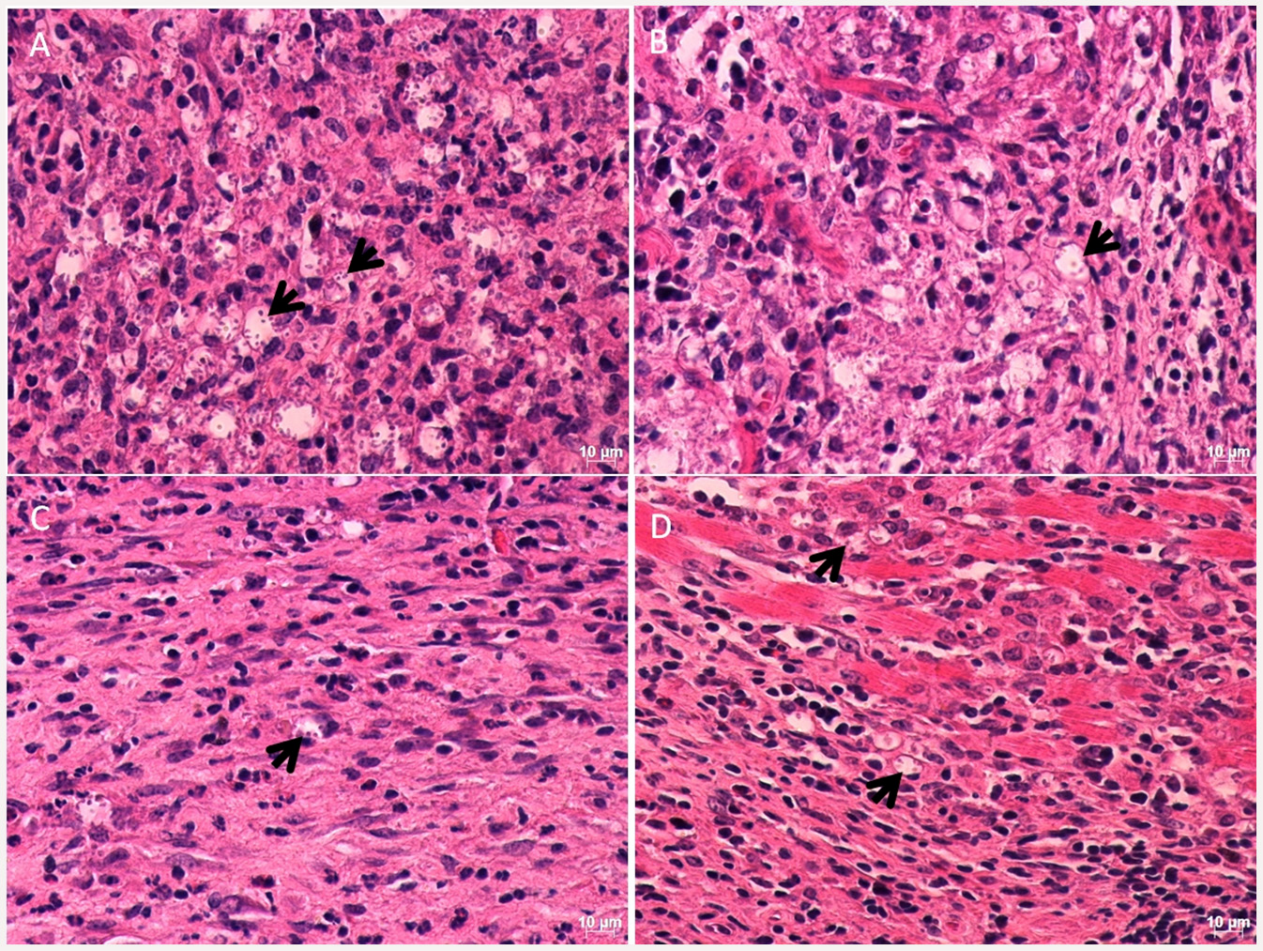
Histological sections of skin from *L*. *(L*.*) amazonensis* mice submitted or not with UA and amphotericin treatments. A—Skin histological section of an infected control mice; B—Skin histological section of an infected BALB/c mice treated with UA (1.0mg/kg); C—Skin histological section of an infected BALB/c mice treated with UA (2.0mg/kg); D—Skin histological section of an infected BALB/c mice treated with amphotericin B (5.0mg/kg). Skin sections were staining with hematoxilin & eosin staining and observed under optical microscope at a magnification of 40x.

Skin of infected non-treated BALB/c mice presented heavily infected vacuolized macrophages (Fig 8A, black arrows). *L*. *(L*.*) amazonensis*-infected BALB/c mice, treated with 1.0 mg/kg of UA also presented vacuolized macrophages with less intracellular amastigotes when compared to control mice (Fig 8B, black arrows). *L*. *(L*.*) amazonensis*-infected BALB/c mice, treated with 2.0 mg/kg of UA showed more preserved epidermis and dermis, and few parasitized macrophages were found (Fig 8C, black arrows). Infected animals treated with amphotericin B also presented few infected vacuolized macrophages compared to non-treated animals, and the majority of these cells were found in deep dermis (Fig 8D, black arrows).

## Discussion

Classic drugs used to treat leishmaniasis, such as antimonials and amphotericin B, are considered obsoletes and most importantly toxic to patients, justifying the rational search for new anti-leishmanial compounds. In this regard, plant extracts, fractions and purified compounds have been extensively assayed in different types of experiments targeting to find more effective and lesser toxic drugs [38]. Some studies already recognized that some triterpenes possess leishmanicidal effects [39], such as OA and UA that present low toxicity into different experimental models [40, 41], proposing these compounds as interesting prototype drugs. In this regard, bioguided-fractionation of acetone extract of the roots of *Salvia cilicica* afforded the purification of UA and OA, being the UA the most active compound against *L*. *major* promastigotes [29]. *Pourouma guianensis* isolated UA showed significant activity against *L*. *(L*.*) amazonensis* promastigotes with an EC_50_ of 5.0 μg/mL [30]. Moreover, UA isolated from *Baccharis dracunculifolia* was effective against *L*. *donovani* promastigotes with an EC_50_ of 3.7 μg/mL. In the present study, purified UA was active against *L*. *(L*.*) amazonensis* promastigotes, presenting an EC_50_ of 6.2 μg/mL, by the other side, its isomer presented only a marginal leishmanicidal effect at 100 μg/mL (~14% of parasite inhibition), as also shown by Tan and collaborators [29]. These data suggest that UA is highly effective against *L*. *(L*.*) amazonensis* promastigotes, and this activity was similar to that induced by the standard drug Miltefosine. In addition, the cytotoxicity induced by UA was similar to amphotericin B and higher than Miltefosine, that in turn correlated with the selectivity indexes, demonstrating that UA presented a marginal cytotoxic effect. In fact it was comparable with amphotericin B and similar to Miltefosine, suggesting that UA can be considered as a prototype drug. Furthermore, studies have demonstrated that promastigote sensitivity and cell toxicity to new compounds frequently indicate how specific a given molecule attacks the parasites and not host cells, as indicated in Table 1, through the selective index. In this case, UA was two times more effective than Miltefosine, but *in vitro*, amphotericin B was more effective.

Although UA represents a prototype drug, studies did not reveal the action mechanism of this triterpene on *Leishmania* sp. parasites. The ultra-structural study revealed that UA caused parasite morphological alteration, which can be associated with apoptosis or even autophagy process [42]. Studies dealing with anti-tumoral effect of UA were able to detect that UA-treated R-HepG2 [43] and SW480 [44] tumoral cells presented ultra-structural alterations similar to that found in UA-treated *L*. *(L*.*) amazonensis*, suggesting that, at least one of the possible mechanism of action triggered by UA is programed cell death. Besides ultra-structural alterations of UA-treated promastigotes, detected by TEM, other phenotypic changes can be associated to programmed cell death in parasites, as is the case of phosphatidylserine exposure from the inner side to the outer layer of the plasma membrane stained by Annexin V [45]. As stated above, UA-treated *L*. *(L*.*) amazonensis* promastigotes presented morphology associated to a process of apoptosis-*like*, that was confirmed trough the detection of two populations by flow cytometry, one Annexin V^+^/PI^-^ (early apoptosis) and the other was Annexin V^+^/PI^+^ (late apoptosis), suggesting that UA triggered programmed cell death in *L*. *(L*.*) amazonensis* promastigotes. Studies conducted with other types of natural compounds, such as saponin racemoside A [46], polyphenol curcumin [47], lignan yangambin [48] and monoterpene citral [49], also induced apoptosis in *L*. *(L*.*) donovani*, *L*. *(L*.*) chagasi* and *L*. *(L*.*) amazonensis* promastigotes. In the present study, in the UA-treated *L*. *(L*.*) amazonensis* group, approximately 36% of the parasites underwent apoptosis and a minor population (~10%) was PI^+^, suggesting that this parasite population also presented loss of membrane integrity, leading to a process of death associated to necrosis [46, 50].

In order to evaluate if apoptosis was dependent or independent of capases, enzymatic reactions were carried out using caspase 3/7 substrates. These enzymes are considered to be an executioner caspases [51], and for this reason, their activities were studied. In this regard, it was observed a slight increase in the activity of caspase 3/7 in UA-treated parasites in comparison with control parasites, and when compared to H_2_O_2_, a positive control for apoptosis, it could be even considered basal. In addition, the activity of caspase from UA-treated parasites was not enough to fragment genomic DNA as caspases from H_2_O_2_-treated parasites did. Considering these findings, programed cell death triggered by UA in *L*. *(L*.*) amazonensis* parasites can be considered independent of caspase. In fact, the majority of promastigote forms treated with UA were in early stage of programmed cell death, that can be firstly characterized by alterations in mitochondria physiology. In Jurkat cell lineage and lymphocytes, an elevation of mitochondrial membrane potential occurs before activation of caspases, and this fact appears to be the earliest change associated with apoptosis pathways (Perl et al 2004). Indeed, UA-treated promastigotes showed an increasing in JC-1 aggregates that can be associated to a hyperpolarized state of mitochondria [52, 53], reinforcing that UA-treated parasites were in an early stage of programmed cell death. Therefore, UA triggered an early event of programmed cell death in *L*. *(L*.*) amazonensis* and this fact was associated with a hyperpolarized state of mitochondria rather than caspase 3/7 activity.

Some evidences have shown that nitric oxide is the major antileishmanial compound produced by macrophages to eliminate intracellular amastigotes of *L*. *(L*.*) amazonensis* infection [54, 55, 56]. In addition, NO can be triggered by different stimuli as well as natural compounds from plants [57]. In the present study, infected peritoneal macrophages treated with UA produced elevated amounts of NO at 24 and 72h compared to controls. Previously, You et al [58] reported that UA triterpene stimulated both peritoneal macrophages and RAW 264.7 cell lineage to produce NO in a dose-dependent manner. Elevation in levels of NO also was observed in J774 treated with a fraction purified from *Baccharis uncinella* leaves that was enriched in UA [25]. Thus, UA can be considered an immunomodulatory compound, since *L*. *(L*.*) amazonensis*-infected peritoneal macrophages produced high levels of NO that could account for UA leishmanicidal activity. Taken together, in this study UA was active against intracellular amastigote and triggered NO production in infected peritoneal macrophages.

In addition to *in vitro* assays conducted with the triterpenes, *in vivo* experiments have been successfullly performed with this class of secondary metabolites. Studies conducted by Germonprez and coauthors [59] demonstrated that triterpenoid saponins purified from *Maesa balansae* were able to reduce liver parasitism in *L*. *(L*.*) infantum*-infected BALB/c mice. In *L*. *donovani*-infected hamster the therapeutic potential of the triterpene saponin maesabalide III was comparable with liposomal amphotericin B [60]. A fraction enriched with UA and OA purified from *Baccharis uncinnela* leaves was therapeutic for *L*. *(L*.*) amazonensis*-infected BALB/c mice, and did not present toxicity for internal organs of mice [31]. Similarly, in the present study, *L*. *(L*.*) amazonensis*—infected BALB/c mice submitted to UA treatment presented lesser lesion size and skin parasitism compared to control-infected mice. Although UA presented therapeutic effect in infected BALB/c mice, Glucantime treatment presented a slight efficacy in experimental disease compared to UA, being able to eliminate around 99.9% of parasite with a total of 10.0 mg administrated via intralesional route, while the treatment with 2.0 mg/kg of UA eliminated around 98% of parasites with a total of 176 μg of UA injected intralesionally. In fact, this is a significant difference between drugs requirement to eliminate intracellular parasites, therefore, it was necessary around 57 times more antimony than UA to eliminate a similar amount of parasites. Interestingly, in the site of parasite inoculation, this decrease in parasitism was also observed by histology, since UA- and Glucantime- treated animals presented lesser vacuolized and infected macrophages, confirming the efficacy of natural and standard drugs. These findings suggest that UA presents a superior efficacy in the experimental treatment of leishmaniasis in comparison with glucantime. Noteworthy, the standard drug Glucantime presents serious side effects for patients, such as fever, gastrointestinal toxicity (e.g. vomiting and abdominal pain) and in more severe cases, cardiotoxicity. By the other side, studies already showed that UA presents low/tolerable or even absent toxicity for different experimental models, and for this reason, it has been used for different clinical applications, including cosmetic industry [61, 62].

In overall the present showed for the first time, that the action mechanism of UA was related to programmed cell death, as evaluated through morphological and biochemical studies; and importantly UA presented therapeutic effect for *L*. *(L*.*) amazonensis*-infected BALB/c mice, suggesting that this triterpene can be considered an interesting candidate for future tests as a prototype drug for the treatment of cutaneous leishmaniasis.

## Supporting Information

S1 File Raw data used in the analysis. (XLSX)
